# Tumor microenvironment in a minipig model of spinal cord glioma

**DOI:** 10.1186/s12967-023-04531-7

**Published:** 2023-09-27

**Authors:** Muhibullah S. Tora, Stewart G. Neill, Yuliya Lakhina, Hemza Assed, Michelle Zhang, Purva P. Nagarajan, Thais Federici, Juanmarco Gutierrez, Kimberly B. Hoang, Yuhong Du, Kecheng Lei, Nicholas M. Boulis

**Affiliations:** 1 Department of Neurosurgery, Emory University School of Medicine, Atlanta, GA USA; 2 Department of Biomedical Engineering, Georgia Institute of Technology, Atlanta, GA USA; 3 Department of Pathology and Laboratory Medicine, Emory University School of Medicine, Atlanta, GA USA; 4 Department of Pharmacology and Chemical Biology, Emory Chemical Biology Discovery Center, Emory University School of Medicine, Atlanta, GA USA

**Keywords:** Minipig model, Spinal cord glioma, Tumor microenvironment, Oxidative stress

## Abstract

**Background:**

Spinal cord glioma (SCG) is considered an orphan disease that lacks effective treatment options with margins that are surgically inaccessible and an overall paucity of literature on the topic. The tumor microenvironment is a critical factor to consider in treatment and modeling design, especially with respect to the unresectable tumor edge. Recently, our group developed a high-grade spinal cord glioma (SCG) model in Göttingen minipigs.

**Methods:**

Immunofluorescence and ELISA were performed to explore the microenvironmental features and inflammation cytokines in this minipig SCG model. Protein carbonyl assay and GSH/GSSG assay were analyzed in the core and edge lesions in the minipig SCG model. The primary core and edge cells proliferation rate were shown in vitro, and the xenograft model in vivo.

**Results:**

We identified an elevated Ki-67 proliferative index, vascular and pericyte markers, CD31 and desmin in the tumor edge as compared to the tumor core. In addition, we found that the tumor edge demonstrated increased pro-inflammatory and gliomagenic cytokines including TNF-α, IL-1β, and IL-6. Furthermore, the mediation of oxidative stress is upregulated in the tumor edge. Hypoxic markers had statistically significant increased staining in the tumor core, but were notably still present in the tumor edge. The edge cells cultures derived from SCG biopsy also demonstrated an increased proliferative rate compared to core cell cultures in a xenotransplantation model.

**Conclusions:**

Our study demonstrates heterogeneity in microenvironmental features in our minipig model of high-grade SCG, with a phenotype at the edge showing increased oxidative stress, proliferation, inflammatory cytokines, neovascularization, and decreased but present staining for hypoxic markers. These findings support the utility of this model as a means for investigating therapeutic approaches targeting the more aggressive and surgically unresectable tumor border.

**Supplementary Information:**

The online version contains supplementary material available at 10.1186/s12967-023-04531-7.

## Introduction

Spinal Cord Gliomas (SCGs) account for 4–8% of central nervous system (CNS) tumors, where high-grade lesions have a poor prognosis [1]. Most commonly, gross total resection is not an option in such lesions due to a lack of resectable margins and the risk of worsening morbidity [2]. Importantly, SCG is considered an orphan disease that is overlooked in comparison to its intracranial counterpart whereby human specimens are limited owing to this relative rarity. Consequently, there is a paucity of literature on SCG pathogenesis, intratumoral heterogeneity, and tumor microenvironment despite some recent small retrospective studies [3–6].

While rodent models have been utilized in the preclinical setting for the study of SCG, there are known limitations especially with regard to anatomic constraints, drug delivery, and surgical translatability [7, 8]. In the past, efforts to investigate surgical techniques, drug delivery, and de novo tumor development have depended on spontaneous canine gliomas [9] which are rare and present with ethical and logistical issues. Moreover, SCG is extremely rare in dogs. As such, development of new therapeutic strategies for SCG has been hindered by the lack of a relevant preclinical animal model for understanding the delivery, therapeutic response, efficacy, toxicity, and tumor microenvironment of SCG.

Our group has developed a minipig model of high-grade SCG using lentiviral gene transfer of oncogenic transgenes including platelet derived growth factor beta (PDGF-B), HRAS-G12V, and small hairpin-loop RNA targeting P53 (shRNA-P53) [10] as well a vetted methodology for inducing glioma in prior rodent studies[11–13] and validated this approach in the rat spinal cord [14]. We employed this approach given involvement of the RTK/RAS/PI3K and p14/CDKN2A and P53 pathways in a large majority of high-grade gliomas [10, 11]. This model has histopathologic and radiologic features consistent with human disease processes in a highly penetrant manner [10]. The advantage of utilizing a large animal model is to facilitate translation given the similarity of human's metabolic, oxidative, genetic, and immunologic features to that of the swine [15]. However, the tumor microenvironment and heterogeneity of our model is unknown. In an effort to further characterize and advance preclinical modeling, the present study reports key microenvironmental features of the model, including oxidative stress, vascular markers, and intratumoral heterogeneity between the tumor core and edge.

Among these features is oxidative stress, a well-known contributor to glioma pathogenesis. It is exerted by reactive oxygen species (ROS) that accumulate due to an imbalance between ROS generation and elimination, which can consequently promote glioma growth and progression [16]. In the tumor microenvironment, an interplay between tumor cells and surrounding parenchyma can enable tumor progression by sustaining malignant cells and providing adaptation to restricted oxygen supply [17]. In addition to adaptation to a hypoxic environment, ROS generation can lead to lipid, protein, and DNA damage that can further contribute to glioma pathogenesis. Furthermore, it is well documented that the tumor core in high grade supratentorial gliomas have a hypoxic core and over-expression and stabilization of hypoxia inducible factor-1 (HIF-1α) [18, 19]. Downstream of HIF-1α are hypoxia responsive genes, including glucose transporter 1 (GLUT-1) and CA-IX (Carbonic Anhydrase 9) which are expressed in cells undergoing anaerobic metabolism and demonstrated to be overexpressed in glioblastoma cells [20]. In addition, this hypoxic microenvironment leads to a variety of downstream effects including a pro-angiogenic environment, metabolic reprogramming, and local immunosuppression [21]. Consequently, understanding whether this phenomenon is present in this model is crucial to its translational relevance.

The differences in oxidative stress, region specific factors and subsequent molecular features may be found depending on the geographic location within the tumor including well-reported differences in proliferation [22], inflammatory cytokines [23], and microvascular structure [24, 25]. Certainly, molecular subtypes have been identified from a transcriptomic standpoint in supratentorial high-grade glioma [26, 27] and correlated with characteristic immunohistochemical profiles in regions of hypoxia and hyper-proliferation amongst others [28]. Given these studies in clinical and preclinical models in the brain, it stands to reason that modeling SCG requires robust characterization of factors. This is especially crucial from a clinical standpoint in the spinal cord, whereby the unresectable edge of the tumor is a major cause for recurrence.

In our present study, we compare and contrast features of the tumor core and edge to evaluate differences in redox homeostasis, hypoxic markers, and validate the aggressive proliferation of tumor cells. We further described recapitulation of these features upon evaluation in porcine SCG-derived culture models both in vitro and on xenotransplantation. These data provide the basis for future work employing this large animal model as a platform for translational surgical studies, pharmacotherapeutic studies targeting redox homeostasis, and comparative studies with patient derived samples.

## Materials and methods

### Gӧttingen minipig spinal cord glioma model

Four Gӧttingen minipigs (± 15–20 kg, 8–9 months old) were employed in the present study. The surgical procedures were performed as previously described for administration of oncogenic vectors. Third-generation and VSV-G pseudotyped lentiviral vectors were employed as previously described [10]. Briefly, three individual vector constructs (1 – EF1a-PDGFB-IRES-eGFP, 2 – EF1a-HRAS-G12V-mPlum, and 3 – H6 & U6 shRNA-P53 with PGK-mCherry) were produced for viral packaging at > 10^9 IU/ml in a standard fashion. Animals were monitored using physical examinations at baseline and post-operatively three times per week until endpoint. All experiments were approved by the Institutional Animal Care and Use Committee (IACUC) in coordination with the Division of Animal Resources (DAR) and veterinary staff [10].

### Magnetic resonance imaging (MRI)

Pigs were scanned used a clinical 3 T Trio MRI with an integrated spine coil (Siemens Medical Solutions, Malvern, PA, USA) using standard clinical sequences including T1-weighted, T2-weighted, and T1-weighted post-gadolinium (0.1 mmol/kg intravenous, Multihance, Bracco Diagnostics, Italy). Scans were reviewed and processed using RadiAnt DICOM Viewer (Medixant, Poznań, Poland) [10].

### Nude mice xenograft model

Athymic nude mice were housed and maintained with the protocols approved by the Emory Institutional Animal Care and Use Committee (IACUC). The nude mice are 5–6 weeks old, half male and half female, with eight mice per group, total 16 mice. Pig SCG core and edge cells (3 × 10^6^) in 100 μl of PBS were inoculated subcutaneously into the mice on the dorsal flank. Body weight and tumor growth were recorded once every three days. The total tumor volume (TV) was calculated by the following formula: TV (mm3) = a * b^2^ /2, where "a" is the minimum diameter, and "b" denotes the maximum diameter. The mice were euthanized after three weeks [29].

### Hematoxylin–eosin (H&E) staining and immunohistochemistry (IHC) staining

The minipig SCG tumors and xenograft tumors were fixed overnight with 4% PFA followed by paraffin embedding. H&E staining was performed to evaluate histopathologic features in pig SCG tumors and xenografts. Immunohistochemistry was performed using primary antibodies for Ki67 (ab15580, ABCAM, USA), SOX2 (ab92494, ABCAM, USA), Olig2 (P21954, Invitrogen, USA), NG2 (ab129051, ABCAM, USA), NQO1 (#3187, Cell Signaling Technology, USA), 4-HNE (MA5-27,570, Invitrogen, USA), HIF-1α (PA1-16,601, Invitrogen, USA), GLUT-1 (ab14683, ABCAM, USA), CA-IX (66,243, Proteintech, USA) with Vectastain Kit (PK-8200, Vector Laboratories, Inc., USA). All images were acquired using whole-slide scanning at 40 × magnification (Leica Aperio AT2 Slide Scanner) [14].

### Immunofluorescence staining

Paraffin embedded sections were also stained with primary antibodies for PDGFB (ab23914, ABCAM, USA), HRAS (sc-29, SANTA CRUZ, USA), p53 (#2524, Cell Signaling Technology, USA), Ki67 (ab15580, ABCAM, USA), NQO1 (#3187, Cell Signaling Technology, USA), 4-HNE (MA5-27,570, Invitrogen, USA), Desmin (MA106401, Invitrogen, USA), CD31 (MCA1746GA, Invitrogen, USA), Collagen IV (ab6586, ABCAM, USA) with Opal Detection Kit (AKOYA BIOSCIENCES, USA) and the images were taken by Vectra Polaris Multispectral Imaging System (AKOYA BIOSCIENCES, USA) [29, 30].

### Enzyme-linked immunosorbent (ELISA) assays

Tissue lysates of the core and edge of minipig SCG samples were harvested and quantified for cytokines levels using ELISA Kits, including IL-1β (ab100754, ABCAM, USA), IL-6 (ab100755, ABCAM USA) and TNF-α (88 ab100756, ABCAM, USA) in a standard fashion according to manufacturer specifications [31].

### 8-hydroxy 2 deoxyguanosine (8-OH-dG) Level, Protein Carbonyl (PC) Assay and GSH/GSSG Assay

A8-OH-dG level and ratio of GSH/GSSG allows for evaluation of the status of oxidative stress in the biological systems [32], while protein carbonyl content can measure Oxidative damage to proteins. The tissue lysates of the core and edge of minipig spinal cord glioma samples were harvested and quantified for 8-hydroxy 2 deoxyguanosine Kit (ab201734, ABCAM, USA), GSH/GSSG-Glo™ (Promega Corporation, USA) and Protein Carbonyl Assay Kit (#ab126287, ABCAM, USA) following with manufacturer's assays procedure.

### ATP Assay and Lactate dehydrogenase (LDH) release

Tissue lysates of minipig SCG samples from core and edge locations were evaluated for total ATP and LDH release using ATP Assay Kit (ab83355, ABCAM, USA) and LDH assay kits (J2380, Promega Corporation, USA) according to the protocols and read using a microplate reader (BioTek Instruments Inc., USA) [31, 33].

### Primary cell cultures of minipig SCG Core and edge samples

After sacrificed the minipigs, the tumors were collected from the spinal cord of the animal. Core and edge cell samples were cultured in Dulbecco's Modified Eagle Medium (DMEM), with addition of media components including 10 ng/mL PDGFAA (# 78,095, STEMCELL Technologies, USA),10 ng/mL bFGF (# 78,003.1, STEMCELL Technologies, USA), N2 Supplement (07152, STEMCELL Technologies, USA), 20% FBS and penicillin (100units/mL)-streptomycin (100 μg/mL) (Hyclone, USA) in a humidified incubator with 5% CO_2_ at 37 °C according to a previously described protocol [34].

### Cell proliferation assay

In order to evaluate cell proliferation, core and edge cells were seeded (2 × 10^5^ cells per mL) in growth medium in a 96-well plate and analyzed by MTT Cell Proliferation Assay Kit (ab211091, ABCAM, USA). Briefly, 50 μL of MTT Reagent was added to each well, and the plates were incubated for 3 h at 37 °C. After incubation, 150 µL of MTT Solvent were added to each well. The absorbance at OD 590 nm was recorded using a microplate reader (BioTek Instruments Inc., USA) at specified timepoints [35].

### Statistical analysis

Data visualizations were performed with GraphPad Prism 6 (GraphPad Software Inc., La Jolla, CA, USA) and BioRender (BioRender, Toronto, Canada). The analyses were performed using either Student’s t-test or one-way ANOVA. Significant Difference among groups was assessed as *p < 0.05; **p < 0.01; ***p < 0.001.

## Results

### Establishing minipig SCG model and comparison of core and edge

**Fig. 1:**
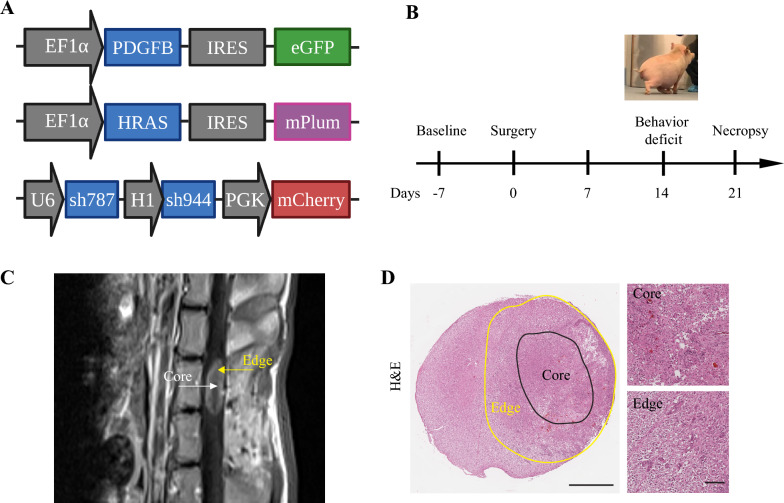
**Establishment a Cohort of Minipig SCG and Defining Core and Edge Regions.** **a** Lentiviral vectors expressing PDGFB, HRASG12V, and shRNA targeting P53 (sh787, sh944) were designed based on prior literature. **b** Schematic representation of the timeline of tumor progression in the spinal cord minipig model. **c** Representative endpoint MRI of T1w post-contrast demonstrating a contrast enhancing lesion in the oncogene injection site. **d** Representative H&E staining showing edge and core tumor regions in the spinal cord glioma. Scale bar: 2 mm and 100 μm

The development of our minipig spinal cord glioma model was reported previously [10]. In short, 14 days post-operatively, animals (N = 4/4) developed motor deficits in accordance with oncogene injection (Fig. 1A and B). 21 days post-operatively, MRI was used to validate the presence of an enhancement mass lesion (Fig. 1C) and animals underwent necropsy for tissue harvesting. H&E was used to confirm histopathologic features including hypercellularity, nuclear atypia, mitotic indices, necrosis, hemorrhage, increased vascularity, and astrocytic morphology. An outline of the definition of core and edge regions of the tumor is presented in Fig. 1D and grossly defined as 1 mm border circumferential border [36].

### Enrichment of SCG edge in pericytes and endothelial cells

**Fig. 2:**
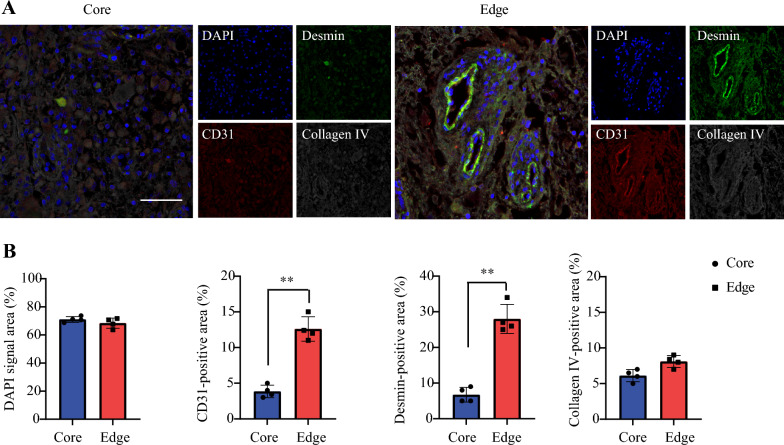
**Vascular Microenvironment of SCG edge Demonstrates Enrichment in Endothelial Cells and Pericytes.** **a** Immunofluorescence imaging of core and edge tumor sections co-stained for anti-CD31(endothelial cells, red), anti-Desmin (pericytes, green), and anti-collagen IV (basement membrane, grey). Scale bars are 50 μm. **b** Quantification of DAPI signal area, CD31, Desmin, and collagen IV-positive areas. Data are shown as means ± SEM. (**p < 0.01, one-way ANOVA, n = 4)

One crucial component to microenvironmental differences in high-grade glioma is differences in vascular structure, including endothelial cells, pericytes, and basement membrane. We co-stained pig SCG tumors with anti-CD31 (PECAM-1), anti-Desmin antibodies, anti–collagen IV antibodies, and DAPI (Fig. 2A). We found a statistically significant elevation in CD-31 and Desmin positivity *(p* < 0.01) in the edge compare to the core cells. No significant difference was observed in the overall cellularity or Collagen IV (Fig. 2B). In the tumor edge, we also observed a high-degree of pericyte coverage of blood vessels and vascular endothelium in a disorganized fashion.

### Increased proliferative glial progenitors and cytokines in the edge

**Fig. 3:**
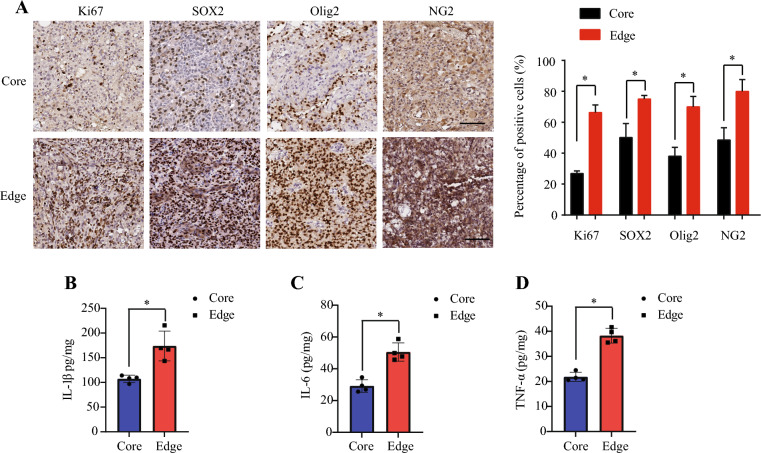
**Tumor Edge Demonstrates Elevated Proliferation and Inflammatory Cytokines.** **a** Glioma markers and proliferative index were analyzed by immunohistochemistry staining. Scale bar: 100 μm. **b** IL-1β, **c** IL-6, and **d** TNF-α levels in the core and edge lysate cells of the minipig SCG model. The spinal cord glioma lysates were subjected to TNF-α, IL-1β, and IL-6 ELISA analysis. Data are shown as means ± SEM. (*p < 0.05, one-way ANOVA, n = 4)

In comparing the tumor core and edge regions, we found statistically significant elevations in proliferative marker Ki-67 as well as glial progenitor markers SOX2, Olig2, and NG2, (*p* < 0.05, Fig. 3A). In order to further characterize gliomagenic cytokines in this model, we evaluated TNF-a, IL-1B, and IL-6. On ELISA of tumor lysates of core and edge samples, edge lysates demonstrated statistically significant elevations in cytokines TNF-α, IL-1β and IL-6 compared to core lysates (Fig. 3 B, C, D).

### Elevated markers of oxidative stress in the SCG edge cells

**Fig. 4:**
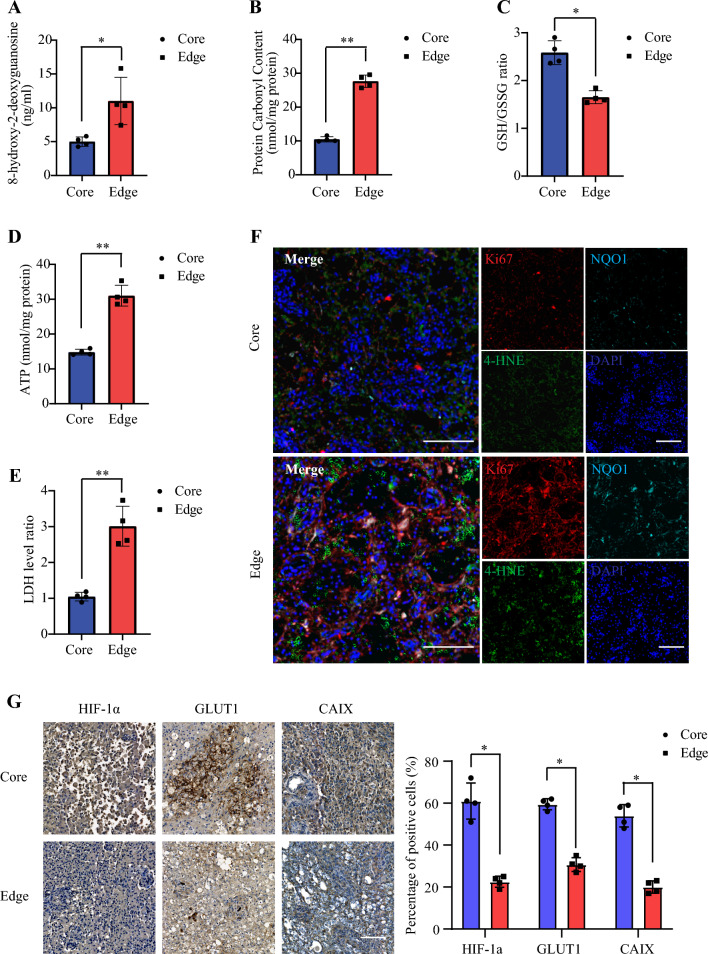
**SCG Tumor Edge Exhibits Increased Markers of Oxidative Stress.** **a** Expression of 8-hydroxy-2-deoxyguanosine. **b** Carbonyl expression levels. **c** GSH/GSSG ratios. **d** ATP levels. **e** LDH ratios. Data are means ± SEM (*p < 0.05; **p < 0.01; ***p < 0.001, n = 4) **f** Representative immunofluorescent staining of edge and core minipig SCG tissues for 4-HNE (green), Ki67 (red), NQO1 (cyan) and DAPI (blue). Scale bars: 100 μm. n = 4. **g** HIF-1α, CA-IX, GLUT-1 staining in tumor core and edge of minipig SCG tissues. Scale bars: 100 μm. n = 4

Given significant elevations in proliferative index, we investigated markers of oxidative stress in the core and edge regions in the tissues. 8-OH-dg, a biomarker of DNA damage in response to oxidative stress, was elevated two-fold on average in edge *versus* core lysates (*p* < 0.05, Fig. 4A). Protein Carbonyl expression levels, a marker of oxidative stress and protein oxidation, were also elevated in the edge samples (*p* < 0.01, Fig. 4B). On examination of GSH to GSSG ratios, core samples were significantly elevated (*p* < 0.05, Fig. 4C) compared to edge samples, indicating increased oxidative stress in the edge samples. Overall, this suggests elevated ROS and oxidative stress in the edge samples compared to core samples. ATP and LDH assay revealed enhanced cell death in edge cells compared to core cells (Fig. 4 D and E). Moreover, 4-HNE and NQO1 mirrored the Ki67 expression patterns on immunofluorescent co-staining (Fig. 4F). Hence, these data support elevated ROS in the edge region of the minipig SCG model. Examining microenvironmental features of redox homeostasis would be incomplete without also characterizing markers of hypoxia in the present model, given its well documented role in glioma progression and impact on glioma cell metabolism [17, 20, 21]. As such, we further examined hypoxic markers through immunohistochemical staining and found that HIF-1α, CA-IX, and GLUT-1 staining was increased in the core compared to the tumor edge (Fig. 4G). This suggests that the present model recapitulates a largely hypoxic tumor core, and that the tumor edge has a degree of hypoxia, albeit less than the tumor core.

### Proliferative phenotype of edge cells is recapitulated in culture and pig-derived xenograft

**Fig. 5:**
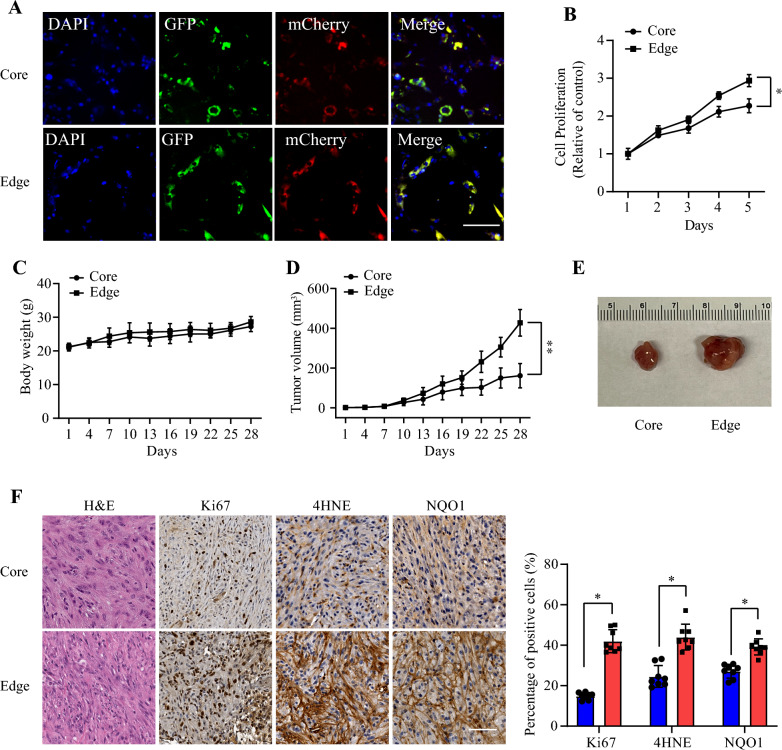
**Cell proliferation of core and edge minipig spinal cord glioma cells in a nude mice xenograft model** **a** Core and edge cells were staining with GFP and mCherry. Scale bar: 50 μm; **b** Cell proliferation rates of two cell lines were determined by MTT assay for 5 days. **c** Body weight of nude mice with flank injection of core and edge cells. **d** Tumor volume curve in nude mice by core and edge cells. **e** Representative macroscopic images of tumor growth in core and edge nude mice models. **f** H&E, Ki67, 4-HNE and NQO1 staining of tumor slices in various groups. Scale Bars: 50 μm. Significant differences are considered when *p < 0.05; **p < 0.01. n = 8

In order to explore whether edge cells recapitulate their proliferative phenotype in vitro, we established the core and edge tissue culture models derived from fresh biopsies of the minipig SCGs collected at the time of euthanasia. We confirmed presence of vector reporters by immunofluorescent staining (Fig. 5A). Next, we compared the rate of proliferation and found that edge cells exhibited higher cell proliferation rates than core cells (Fig. 5B).

We then looked to examine this phenotype in vivo in a xenograft model. Both cell lines were inoculated in nude mice subcutaneously, and tumor volumes were quantitatively measured every three days. The tumor growth did not significantly affect the nude mice's body weight (Fig. 5C). Notably, the edge xenografts exhibited significantly increased tumor growth compared to the core cells (Fig. 5D and E). After termination, H&E staining showed a high-grade glioma with features consistent with prior analyses in both core and edge xenografts. Ki-67 staining once more showed that cell proliferation was strongly elevated in the edge xenografts. ROS staining assay with 4-HNE (4-hydroxynonenal), a marker for ROS level, showed that edge cells evidently ameliorated ROS, coupled with NQO1 immunohistochemical staining (Fig. 5F). We then examined if the xenograft model would reproduce the findings on hypoxic markers, and found similar results on immunohistochemical staining with increased HIF-1α, CA-IX, and GLUT-1 staining in the tumor core vs the tumor edge (Additional file 1: Figure S1).

## Discussion

Our present study corroborated several key features of our previously described minipig model of high-grade SCG. From a practical standpoint, we continued to report a highly penetrant model system with tumor induction following vector administration. We further described and confirmed the expression of relevant transgenes on immunofluorescence with no major differences in the tumor core compared to the tumor edge. Of note, we observed significant elevation in proliferation in the SCG tumor edge. This finding was recapitulated on establishing tissue culture from biopsies of the core and edge, as well as on xenotransplantation into nude mice. This highlights a point of clinical interest, - it is often tumor cells at the periphery which have higher proliferation rates that are surgically inaccessible given the infiltrative nature of eloquent CNS regions like SCG. The inability to perform a gross total resection given the infiltrative nature of SCGs represents one of main barriers to development of an effective treatment strategies and is the key reason for our interest in the heterogeneity between cells in the tumor core and edge.

The findings in our model were in agreement with the literature in describing increased proliferation, increased vascularity, and oxidative stress at the edge in supratentorial glioma as well as increased hypoxic markers in the tumor core [20, 37–39]. It has been widely reported that the tumor edge in supratentorial high-grade glioma exhibits a more aggressive, proliferative, and invasive phenotype [22, 39]. We examined vascular markers and found that edge regions were enriched in endothelial cells, pericytes, and overall vasculature consistent with prior reports [24]. In non-disease states, pericytes have a multitude of roles, including maintenance of the blood–brain-barrier through interaction with astrocytes, development and maturation of vasculature, paracrine signaling, as well as exhibiting stem cell properties [40–43]. It has also been reported that pericytes directly promote glioma progression in in vitro and in vivo models [44]. Moreover, microvascular proliferation is a well-known feature of high-grade gliomas. In order to explain this finding, the increased vascularity at the tumor edge in this model might simply be due to the innate nature of microvascular proliferation secondary to rising metabolic needs locally in the more mitotically active edge region. This proliferation is conventionally attributed to the fact that hypoxia was not necessarily restricted solely to the tumor core [37]. This concept is supported by the fact that while the tumor edge had, as expected, a significantly lower degree of staining for hypoxic markers (HIF-1a, CA-IX, GLUT-1), up to 30% of the cells in the tumor edge still stained strongly for these markers (Fig. 4G, Additional file 1: Figure S1). As such, this region may represent a site of transitional hypoxia in the setting of rapid tumor proliferation that should be examined in future studies including identifying specific cell populations and highlighting potential translational targets [37]. This is of particular interest given that since pericytes and endothelial cells both express PDGF receptors, it is also possible that our minipig model with the PDGFB transgene have been driven to elevated levels of pericytes and microvascular proliferation [24].

In examining select cytokines in the core and edge regions, we observed an elevation in TNF-α, IL-1β, and IL-6 in the edge compared to the tumor core. IL-6 is a cytokine that is known to trigger JAK-STAT3 signaling in glioblastoma, gliomagenesis, MGMT methylation, and is correlated with tumor grade and overall patient survival [45–47]. TNF-α and IL-1β are pro-inflammatory cytokines that are found in supratentorial high-grade gliomas and are involved in neuroinflammation, gliomagenesis, and development of radio-resistance [23, 48]. Overall, the elevated presence of these cytokines throughout the tumor was consistent with reports on supratentorial high-grade gliomas. It was of interest with utilizing this model as a platform for radio-resistance, drug resistance, or aggressive phenotype in this particularly challenging region.

For evaluation of oxidative stress, we examined markers of oxidative DNA damage (8-OH-dg), protein oxidative damage (protein carbonyl content), as well as non-enzymatic antioxidant defense through ROS scavenging (GSH/GSSG ratio) [49]. We found that the tumor edge has a significantly elevated level of markers of ROS, including a high 8-OH-dG and low GSH/GSSG ratio, as well as oxidative damage with an elevated protein carbonyl content in Edge tumor lysates. This supports the notion of elevated oxidative stress in the setting of a highly proliferative tumor edge. On histologic evaluation, we further observed an increase in NQO1 and 4-HNE staining patterns at the tumor edge. Next steps toward further examining molecular characteristics of core and edge regions of this model will include rigorous evaluation of hypoxic markers including , PDK1 and others. In addition to redox mechanisms contribution to glioma pathophysiology [17], the difference in oxidative stress between the core and edge in this model is of particular interest given that there are a variety of druggable targets in the realm of oxidative stress [16, 29, 50–53].

The development of advanced pre-clinical disease modeling has increasingly employed pigs as biomedical models given their increased anatomic, genetic, and immunologic similarity to the human [54]. Porcine models, including minipigs, have successfully been utilized to model numerous pathologies including colorectal cancer, osteosarcoma, cystic fibrosis, muscular dystrophy, as well as numerous others [55, 56]. Notably, the use of TALEN mediated gene editing was utilized to generate a minipig model of neurofibromatosis type 1 that better recapitulates features and natural history of the human disease [10, 57]. Specific to glioma models, as of 2020 there are only three published models including two orthotopic xenografts [58, 59] using commercially available cell lines, as well as our presently reported spinal cord model using vector driven gene transfer [60]. Further work to develop enhanced porcine models of glioma may create a platform better suited for pre-clinical translational efforts.

## Conclusions

Overall, the data presented in this study demonstrated multiple distinct microenvironmental features within the pig model of high-grade SCG. Future work on the development of this model system could explore each of these individual components, including but not limited to profiling the inflammatory microenvironment, transcriptomic characterization, or further elucidating the cause for findings of intra-tumoral heterogeneity between the core, edge, perivascular niche, or other regions. However, at present this model represents a step forward for facilitating surgical translation, as well as a potential space for evaluating proof-of-concept for surgical resection followed by select druggable targets that could prove more efficient in treating the infiltrative tumor edge which is surgically inaccessible. It would be advantageous to also initially employ culture and xenograft models derived from the pig SCG with high-throughput screening methodologies to identify existing FDA-approved or novel candidates that may be able to better target the tumor edge cells.

### Supplementary Information

**Additional file 1: Figure S1**. IHC staining of edge and core groups for HIF-1α, GLUT1 and CAIX in a nude mice xenograft model. Scale bars: 100 μm. Significant differences are considered when *p < 0.05. n = 8.

## Data Availability

The datasets used and/or analyzed during the current study are available from the corresponding author on reasonable request.

## Abbreviations

SCG: Spinal cord glioma
CNS: Central nervous system
ROS: Reactive oxygen species
H&E: Hematoxylin–eosin
IHC: Immunohistochemistry
IF: Immunofluorescence
8-OH-dG: 8-Hydroxy 2 deoxyguanosine
PC: Protein Carbonyl
LDH: Lactate dehydrogenase
4-HNE: 4-Hydroxynonenal
NQO1: NAD(P)H quinone dehydrogenase 1
