# Supplementary figures and images for: Tumor microenvironment in a minipig model of spinal cord glioma
Source: J Transl Med. 2023 Sep 27;21:667. doi: 10.1186/s12967-023-04531-7 (PMC10523785; doi:10.1186/s12967-023-04531-7)

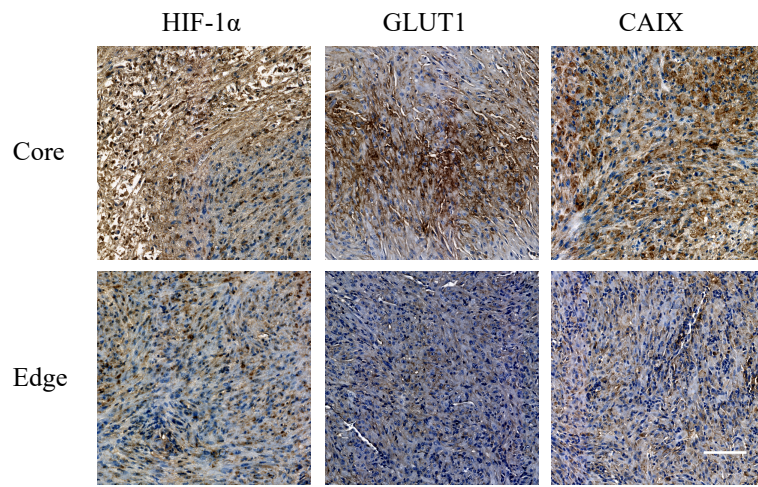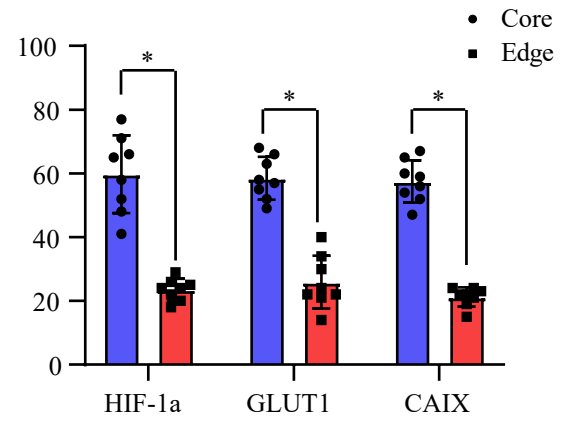

Supplement: Supplementary file 1 — Additional file 1: Figure S1. IHC staining of edge and core groups for HIF-1α, GLUT1 and CAIX in a nude mice xenograft model. Scale bars: 100 μm. Significant differences are considered when *p < 0.05. n = 8. [file 12967_2023_4531_MOESM1_ESM.pdf]
